# Differences of clinical features, prognosis and genetic mutations in Chinese patients with malignant melanoma and additional primary tumours

**DOI:** 10.1080/07853890.2025.2493769

**Published:** 2025-05-03

**Authors:** Jing Lin, Zhongqiao Lin, Yanping Chen, Xuefeng Wang, Yufang Huang, Huishan Zhang, Li Zhu, Zelong Xu, Xuan Gao, Yingqian Zhang, Bin Lan, Yu Chen

**Affiliations:** ^a^ Department of Medical Oncology, Clinical Oncology School of Fujian Medical University, Fujian Cancer Hospital, Fuzhou, China; ^b^ Cancer Bio-Immunotherapy Center, Clinical Oncology School of Fujian Medical University, Fujian Cancer Hospital, Fuzhou, China; ^c^ Phase I Clinical Trial Ward, Clinical Oncology School of Fujian Medical University, Fujian Cancer Hospital, Fuzhou, China; ^d^ Department of Pathology, Clinical Oncology School of Fujian Medical University, Fujian Cancer Hospital, Fuzhou, China; ^e^ Innovation Center for Cancer Research, Clinical Oncology School of Fujian Medical University, Fujian Cancer Hospital, Fuzhou, China; ^f^ Clinical Oncology School of Fujian Medical University, Fuzhou, China; ^g^ Geneplus-Beijing Institute, Beijing, China; ^h^ GenePlus-Shenzhen Clinical Laboratory, Shenzhen, China; ^i^ Laboratory of Radiation Oncology and Radiobiology, Clinical Oncology School of Fujian Medical University, Fujian Cancer Hospital, Fuzhou, China

**Keywords:** Multiple primary malignant tumours, melanoma, epidemiology, prognosis, genetic mutation

## Abstract

**Background:**

The differences in the clinical features, prognosis and genetic mutations in Chinese patients with malignant melanoma (MM) and additional primary tumours remain unclear.

**Methods:**

A retrospective analysis was conducted on patients with malignancies in Fujian Cancer Hospital from January 2007 to September 2022, end follow-up in September 2023. Clinical data were gathered, survival analysis was performed, and genetic mutations were detected.

**Results:**

There were 58 of 1223 melanoma patients with melanoma and additional primary tumours, an incidence of 4.74%. Acral MM was the most common subtype (26/58), 23 (39.66%) patients had concomitant digestive tumours. Patients who had MM as their first primary tumour (MMFP) had shorter tumour occurrence intervals (9.93 vs. 57.78 months, *p* = .008) but longer melanoma survival (MM-OS) than the non-MMFP group (100.43 vs. 18.93 months, *p* = .015). Patients with cancer family histories were more likely to have pathogenic and likely pathogenic (P/LP) mutations (2/5 vs. 4/25). The somatic BRAF gene mutation was frequently observed in MM tissue (8/19, 42.11%). Three patients had whole-genome doubling and microsatellite instability-high (MSI-H). The COSMIC2 signature 3 was significantly higher in the P/LP group.

**Conclusions:**

The frequency of MM and additional primary tumours is about 5% in Chinese populations. Patients with melanoma diagnosed first have longer melanoma survival. Digestive system tumours were the most concomitant; a digestive examination is advisable, especially for those with an expected overall survival (OS) greater than 10 months. Meanwhile, patient’s family cancer history should be followed up in detail, along with completion of germline P/LP mutation and somatic mutation testing, all of which may provide valuable support for further treatment.

## 1. Introduction

Multiple primary malignant tumours (MPMTs) are defined as two or more primary malignant tumours with different origins or pathological types occurring in the same individual [1]. It has been reported that the incidence of multiple primary tumours ranges from 2.4% to 8% [2]. A study from the SEER (The Surveillance, Epidemiology and End Results) program reported that patients having malignant melanoma (MM) as the first primary malignance would have a 10% risk of growing a second primary malignance, with standard incidence rates of 1.11 to have breast cancer or prostate cancer as the second primary malignance [3]. In addition, black patients have a lower incidence of MPMTs and lower survival rates than white patients [4]. Therefore, the characteristics of MPMTs are also different in different races. However, there have been no studies on the epidemiology, clinical characteristics and survival time of patients with melanoma combined with a second primary tumour in a Chinese cohort.

The specific mechanism of MPMTs is still uncertain. Genetic mutations and hereditary components appear to be involved in the occurrence and development of tumours and they can affect patient outcome. Women with two or more relatives having a history of breast cancer have a 2.5-fold increased risk of developing breast cancer and a more positive ER/PR status [5,6]. Colorectal cancer has heritable components that are estimated at about 15–30% [7], and young patients (<50 years old at diagnosis) with a positive family history had less advanced disease and longer survival [8]. More interestingly, the PLCO (The Prostate, Lung, Colorectal and Ovarian) trial revealed that female participants with a family history of prostate cancer in first-degree relatives (FDRs) were more likely to develop postmenopausal breast cancer [9]. Our knowledge of family histories of cancer and the hereditary risk in patients with MM and other primary tumours is inadequate.

Germline mutations and somatic mutations are common and variable in oncological diseases, which may lead to the occurrence and contribute to the pathogenesis of MPMTs. Mutations in BRCA1 and BRCA2 are associated with high risks of second primary breast cancer or ovarian cancer [10]. PALB2 gene variations are associated with increased risk of second primary ovarian or stomach carcinomas [11,12]. In patients with melanoma, wide attention has been attached on the PI3K/AKT/NF-κB pathway, as the AKT family member mutations are often dysregulated in melanoma and PTEN, which classically dampens the PI3K/AKT/mTOR growth-promoting signalling cascade, is noted in nearly one-half of melanoma patients [13,14]. Changes in PTEN and BRAF pathways are often concomitant and associated with resistance to BRAF inhibitors in metastatic melanoma [15]. Besides, germline CDKN2A mutations were also notable for their highly genetic susceptibility in younger patients with multiple primary melanomas [16]. But for patients with MM and other primary tumours, potential germline mutant genes and systemic mutant genes are not clearly defined and are worth determining.

Genomic instability may also play an important role in MPMTs. The microsatellite instability (MSI) phenotype has been associated with the development of multiple primary gastrointestinal cancers in several studies, and might be a useful marker for predicting MPMTs in high risk patients [17–19]. The expression of immune cell PD-L1 was significantly higher in MSI-high (MSI-H) colorectal cancers compared to MSI-low tumours, while there was no difference between the different MSI-H molecular subtypes [20]. Current study showed immunohistochemical testing of mismatch repair mechanisms may yield different results for a given germline mutation, which may be due to somatic mutations [21]. Hence, it is still challenge in distilling the biological and technical heterogeneity of MSI testing down to usable data. However, the MSI and HRD status in patients with MM and additional primary tumours are not known at present. Besides, the genetic differences between patients with MM and additional primary tumours and patients with MM single primary tumours are also worth exploring.

In this study, patients with MM and additional primary tumours were enrolled and their clinical data and prognosis were collected and analysed. The genetic mutations and genomic instability of tumour samples were examined to fully describe the clinical features and genetic alterations, and to look for specific mutations that lead to the additional primary tumours.

## 2. Materials and methods

### 2.1. Patient sources, grouping and clinical data

This retrospective analysis was conducted on patients with melanoma and additional primary tumours, who were admitted to Fujian Cancer Hospital from January 2007 to September 2022. The follow-up period ended in September 2023. The IACR/IARC (International Agency for Research on Cancer) rules were followed as the criteria for diagnosis of MPMTs: (1) cancer occurs at different or at same origin but with different histologic types than the first malignant tumour diagnosed, which should be considered as a separate primary tumour and (2) a 6-month period was used to distinguish between synchronous and metachronous MPMTs (SMPC or MMPC). Patients with malignancies were diagnosed through detailed medical history, complete physical examination and pathologic results. The enrolled patients must meet the following requirements: (1) one of the malignant tumours must be melanoma and (2) the concomitant malignant tumours should except melanoma. A total of 58 patients were included and the clinical data, including tumour family history (TFH) and survival, were collected, and analysed. There were 30 patients from whom we obtained normal tissue samples and detected germline mutations, and 19 of them had complete somatic mutation sequencing of melanoma samples. Another 327 patients with only melanoma were matched by age from the Geneplus database. The data of germline mutations were gathered from Geneplus, and we compared the differences with our data. This study complied with the standards of the Declaration of Helsinki and was approved by the IRB of Geneplus-Beijing and the Ethics Committee of Fujian Cancer Hospital (K2023-246-01). Informed consents were obtained from all patients or their legal guardians in verbal forms during telephone survival follow-up visits. The verbal informed consents were documented by recorded telephone call, in which the patient and their legal guardians had given permissions for the calls to be recorded. The Ethics Committee of Fujian Cancer Hospital approved verbal consent.

The levels of TFHs were defined as follows: (1) first-level family history includes parents, brothers, sisters and children; (2) the second-level family history includes grandparents, aunts, uncles, grandchildren, nieces and nephews. The classifications of hereditary factors were defined as follows: (1) extremely high-risk: ≥3 first-level family members or family history of MM; (2) high risk: two first-level family members; (3) medium risk: one first-level family members or only second-level family members [22–24].

Age cut-offs were divided into: <60 years old, 60–80 years old and >80 years old, according to the guideline [25]. The survival time of malignant melanoma (MM-OS) was calculated as the number of months from the date of MM diagnosis to the date of death or the date of the end of the follow-up (September 2023). The melanoma-specific survival (MSS) time is calculated as the number of months from the date of diagnosis of MM to the date of death caused by MM.

### 2.2. Genetic mutations

The whole exome sequencing (WES) assay was used to determine germline mutations and somatic mutations of tumour samples in patients with MM and additional primary tumours. DNA extracted from fresh tissue samples and blood was sequenced using the HiSeq X platform (Illumina, San Diego, CA) to detect somatic mutations and germline mutations, respectively. After removing low-quality and short reads, trimming adapters from raw reads, clean reads were left and aligned to the human reference genome (hg19) and deduplicated. Local realignment around single nucleotide variants (SNVs) and insertions and deletions (InDels), as well as quality control assessment, were performed next.

For somatic mutations, after somatic SNVs and small InDels were filtered by defined criteria, tumour mutation burden (TMB), MSI and tumour neoantigen burden (TNB) were calculated. The analysis of mutational signature characteristics was conducted using an R package *deconstructSigs* (version 1.8.0). This was based on the identified copy number variation (CNV), tumour purity, ploidy, whole-genome doubling (WGD) and homologous recombination deficiency (HRD). For germline mutations, pathogenic and likely pathogenic (P/LP) variants were annotated by InterVar software, which was established based on the ACMG 2015 criteria provided by the ACMG guidelines [26]. For further detailed information on the next generation sequencing, the process of mutations filtering, and characteristic calculation, see the supplementary method.

### 2.3. Statistical analysis

Data were analysed by SPSS version 26.0 (IBM, Armonk, NY) and R version 3.6 (The R Foundation, Vienna, Austria). Continuous variables are presented as the mean ± SD when normally distributed and as the median (interquartile range) otherwise. Descriptive parameters such as frequencies and percentages were calculated for categorical data. For normally distributed continuous variables, between-group differences were performed with independent-sample *t*-tests; for non-normally distributed data, Wilcoxon’s rank-sum tests were used. All tests were two-sided, and an alpha value of 0.05 was used to define statistical significance.

The survival time of different groups was calculated and the comparisons were evaluated by Kaplan–Meier’s curves and log-rank statistics. All survival data were right-censored. *p* Value <.05 indicated statistical significance.

## 3. Results

### 3.1. Clinical features and survival in patients with malignant melanoma and additional primary tumours

During the period from January 2007 to September 2022, there were a total of 1223 patients diagnosed with MM in Fujian Cancer Hospital. Among them, there were 58 patients who had MM and additional primary tumours, a frequency of 4.74%. These 58 patients were included for an overview of clinical features (Tables 1 and 2). Generally, acral MM was most common among the 58 patients (26/58), followed by mucosal MM (13/58), cutaneous MM (10/58) and unknown primary (9/58). The median age at the diagnosis of the first primary tumour was 57 years old, and the median age for melanoma was 60 years old. Nearly, half of the patients (25/58) were diagnosed with MM at clinical stage I, and advanced MM (stage IV) was diagnosed in 17 of 58 patients. Concomitant digestive system tumours were the most common type seen in the 58 patients (21/58), followed by respiratory system tumours (13/58), and thyroid tumours (11/58). The median MM-OS of the 58 patients was 43.03 months. Patients were further divided into different subgroups based on shared properties.

**Table 1.** Overview of clinical features in patients with MM and additional primary tumours.

Variable	Overall (*n*)	MMFP group (*n*, %)	Non-MMFP group (*n*, %)	*p* Value
Patients	58	28	30	
Gender				.611
Male	27	13 (48.15)	14 (51.85)	
Female	31	15 (48.39)	16 (51.61)	
Status				–
Alive	25	14 (56.00)	11 (44.00)	
Dead	33	14 (42.42)	19 (57.58)	
Age when FPC diagnosed, years				.895
<60	35	18 (51.43)	17 (48.57)	
60–80	22	9 (40.91)	13 (59.09)	
>80	1	1 (100.00)	0 (0.00)	
Age when MM diagnosed, years				.021
<60	28	18 (64.29)	10 (35.71)	
60–80	27	9 (33.33)	18 (66.67)	
>80	3	1 (33.33)	2 (66.67)	
Number of cancer type				.800
2	52	26 (50.00)	26 (50.00)	
3	5	1 (20.00)	4 (80.00)	
4	1	1 (100.00)	0 (0.00)	
Occurrence				.152
SMPC	12	10 (83.33)	2 (16.67)	
MMPC	46	18 (39.13)	28 (60.87)	
Primary location of MM				.377
Cutaneous	10	8 (80.00)	2 (20.00)	
Acral	26	13 (50.00)	13 (50.00)	
Mucosal	13	4 (30.77)	9 (69.23)	
Unknown primary	9	3 (33.33)	6 (66.67)	
MM clinical stage				.466
I	25	12 (48.00)	13 (52.00)	
II	7	5 (71.43)	2 (28.57)	
III	9	4 (44.44)	5 (55.56)	
IV	17	7 (41.18)	10 (58.82)	
BRAF mutation				.543
V600E mutation	8	4 (50.00)	4 (50.00)	
Wild type	17	10 (58.82)	7 (41.18)	
Undetected	33	14 (42.42)	19 (57.58)	
Treatment of melanoma				–
Surgery	46	26 (56.52)	20 (43.48)	
Chemotherapy	9	3 (33.33)	6 (66.67)	
Target/immunological	27	12 (44.44)	15 (55.56)	
Sites of concomitant tumours				.537
Head and neck	5	2 (40.00)	3 (60.00	
Thyroid	11	7 (63.64	4 (36.36)	
Respiratory system	13	6 (46.15)	7 (53.85)	
Digestive system	21	938.10)	1261.90)	
Urogenital System	10	3 (30.00)	7 (70.00)	
Breast	2	0 (0.00)	2 (100.00)	
Others	2	1 (50.00	1 (50.00)	
Death reason				.338
Melanoma	20	7 (35.00)	13 (65.00)	
Other	13	7 (53.85)	6 (46.15)	
Family history				.434
Yes	12	7 (58.33)	5 (41.67)	
No	46	21 (45.65)	25 (54.35)	
Genetic risk classification				.369
No family history	46	21 (45.65)	25 (54.35)	
Extremely high	4	1 (25.00)	3 (75.00)	
High	3	2 (67.00)	1 (33.00)	
Median	5	2 (40.00)	3 (60.00)	

**Table 2.** Differences in age, interval time and survival in patients with MM and additional primary tumours.

	Overall	MMFP group	Non-MMFP group	*p* Value
Age when FPC diagnosed, years				.376
Median	57.00 (interquartile range: 50.75–65.25)	56.50	58.00	
Age when MM diagnosed, years				.437
Median	60.00 (interquartile range: 53.00–67.25)	56.5	63.00	
Interval of the occurrence, months				.008
Median	29.84 (95%CI 7.71–45.03)	9.93 (95%CI 3.07–16.39)	57.78 (95%CI 29.04–86.02)	
MM-OS, months				.015
Median	43.03 (95%CI 33.80–52.27)	100.43 (95%CI 13.60–187.26)	18.93 (95%CI 0.00–41.71)	
MSS, months				.017
Median	12.27 (95%CI 0.00–25.48)	29.97 (95%CI 2.77–57.17)	11.50 (95%CI 7.31–15.69)	

**Figure 1.**
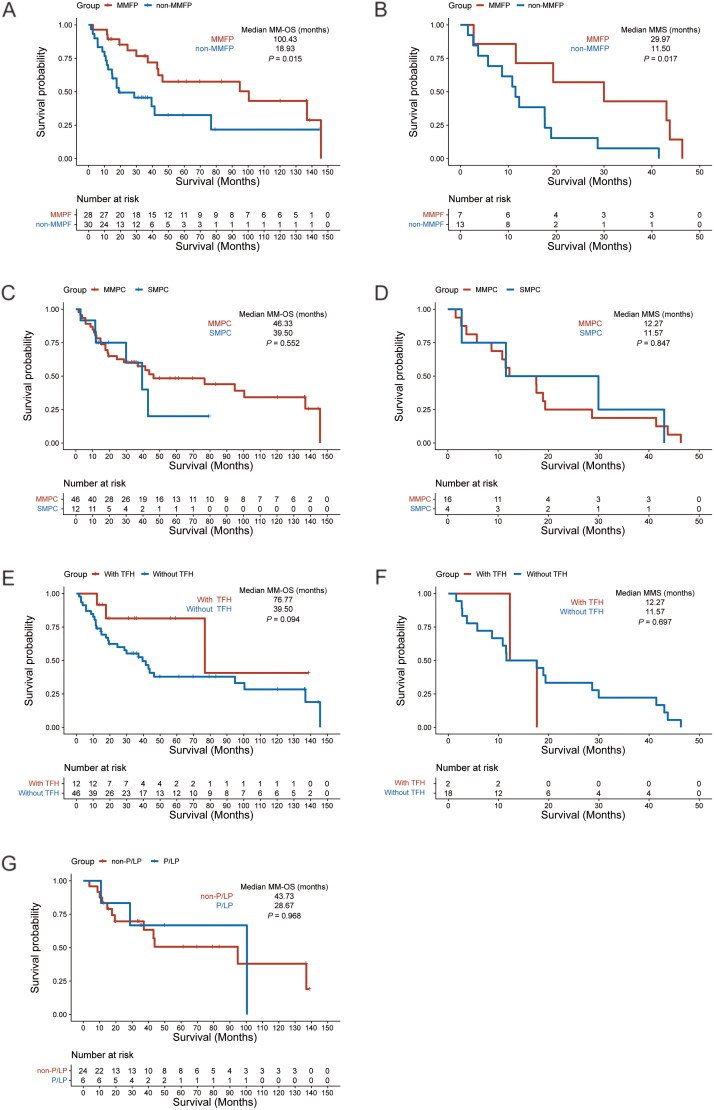
Survival differences in patients with melanoma and additional primary tumours. (A) The survival time of malignant melanoma (MM-OS) in MMFP patients and non-MMFP patients. (B) The melanoma-specific survival time (MSS) in MMFP patients and non-MMFP patients. (C) MM-OS in SMPC patients and MMPC patients. (D) MSS in SMPC patients and MMPC patients. (E) MM-OS in patients with or without tumour family histories. (F) MSS in patients with or without tumour family histories. (G) MM-OS in patients with P/LP or non-P/LP germline mutation.

As shown in Tables 1 and 2, in patients who had MM as their first primary tumour (MMFP) most were younger than 60 years old, younger than those (non-MMFP) first diagnosed with other primary tumours (*p* = .021). Acral MM was the most prevalent in both groups, with 13 patients in each group. Thyroid tumours were the second most common concomitant tumours in the MMFP group (7/28), while in the non-MMFP group, respiratory and urogenital tumours were the second most common concomitant tumours (both 7/28). Patients in the MMFP group also had markedly shorter interval time between the occurrence of MM and other tumours than patients in the non-MMFP group (median time 9.93 vs. 57.78 months, *p* = .008). Although patients in the MMFP group had a short interval before second tumour diagnosis, the MM-OS was markedly longer than non-MMFP group (median time 100.43 vs. 18.93 months, *p* = .015) (Figure 1(A)). Furthermore, the longer MSS time was observed in the MMFP group than in the non-MMFP group (median time 29.97 vs. 11.50 months, *p* = .017) (Figure 1(B)).

Among the 58 patients, there were 12 patients who belonged to the SMPC group and 46 patients who belonged to the MMPC group (Supplementary Tables 1 and 2). The eight patients with BRAF mutations were all found in the MMPC group. After digestive tumours, thyroid tumours were the second most common concomitant tumours in the SMPC group (3/12), whereas 11/46 respiratory tumours were the second most prevalent tumour in the MMPC group (11/46). In the SMPC group, nearly all the patients had MMFP and more than 50% (7/12) were diagnosed with advanced MM (stage III and stage IV). Therefore, shorter MM-OS (median time 39.50 vs. 46.33 months, *p* = .552) and MSS (median time 12.27 vs. 11.57 months, *p* = .847) were observed in the SMPC patients (Figure 1(C,D)).

It was noticed that 12 of 58 patients had TFHs. Among them, 6/12 patients were diagnosed first with MM, and 6/12 had acral MM (Supplementary Tables 3 and 4). There were 3/12 patients who belonged to the SMPCs group for tumour occurrence. Age at first primary tumour diagnosis and clinical stage of MM showed no statistical differences between patients with and without family histories of cancer. Only first-degree family cancer histories were found in 9/12 patients and a second-degree family history was found in one patient. The other two patients had both first-degree family histories and second-degree family histories of cancer. Hereditary risk stratifications were graded by the number of family members with a history of cancer. There were four patients with first-degree family cancer histories who were classified as extremely high risk (≥3 family members had tumours). Overall, patients with family histories of cancer had delayed onset of their first primary tumour and MM, and both MM-OS and MSS were longer than patients without a family history of cancer (median MM-OS was 76.77 vs. 39.50 months *p* = .094, and the median MMS was 12.27 vs. 11.57, *p* = .697) (Supplementary Table 4, Figure 1(E,F)).

### 3.2. Differences on germline mutations in patients with MM and additional primary tumours

There were 30 patients with MM and additional primary tumours who completed germline mutation detection. Six of them were found with germline P/LP mutations and 3/6 patients belonged to the SMPC group. As shown in Tables 3 and 4, the majority of patients in both groups were diagnosed with their first primary tumours or MM before the age of 60 years old, while statistically, patients in the P/LP group seem to have earlier onset of tumours; median age of first primary tumours was at 47.50 vs. 54.50 years old (*p* = .631) and median age of melanoma was at 47.50 vs. 56.50 years old (*p* = .190). Among these 30 patients, five patients had family histories of cancer. Those with cancer family histories were more likely to have P/LP mutations (2/5 vs. 4/25, Supplement Table 5). In the 30 patients with a family history, there was a higher tendency to have concomitant thyroid tumours (*p* = .020, Supplementary Table 5). Furthermore, a shorter median MM-OS was also observed in the P/LP group, but without a significant difference (28.67 vs. 43.73 months, *p* = .968) (Figure 1(G)).

**Table 3.** Overview of clinical features in patients with MM and additional primary tumours under conditions of germline mutations.

Variable	Overall (*n*)	P/LP group (*n*, %)	Non-P/LP group (*n*, %)	*p* Value
*Patients*	30	6	24	
Gender				.378
Male	16	2	14	
Female	14	4	10	
Status				–
Alive	11	4	7	
Dead	19	2	17	
Age when FPC diagnosed, years				.418
<60	25	5	20	
60–80	5	1	4	
Age when MM diagnosed, years				.190
<60	22	5	17	
60–80	8	1	7	
First primary cancer				.651
Melanoma	15	4	11	
Other	15	2	13	
Number of cancer type				.855
2	25	5	20	
3	4	1	3	
4	1	0	1	
Occurrence				–
SMPC	6	1	5	
MMPC	24	5	19	
Primary location of MM				.791
Cutaneous	6	1	5	
Mucosal	16	4	12	
Acral	5	1	4	
Unknown primary	3	0	3	
MM clinical stage				.368
I	16	5	11	
II	7	1	6	
III	2	0	2	
IV	5	0	5	
BRAF mutation				.659
V600E mutation	6	1	5	
Wild type	6	2	4	
Undetected	18	3	15	
Treatment of melanoma				–
Surgery	26	5	21	
Chemotherapy	5	0	5	
Target/immunological	17	5	12	
Sites of concomitant tumours				.306
Head and neck	4	2	2	
Thyroid	9	3	6	
Respiratory system	6	1	5	
Digestive system	11	2	9	
Urogenital system	5	1	4	
Breast	1	0	1	
Death reason				.113
Melanoma	10	0	10	
Other	9	2	7	
Family history				.254
Yes	5	2	3	
No	25	4	21	
Genetic risk classification				.118
No family history	25	4	21	
Extremely high	2	1	1	
High	1	1	0	
Median	2	0	2	

**Table 4.** Differences in age, interval time and survival in patients with MM and other additional primary tumours under the conditions of P/LP germline mutations.

	P/LP	Non-P/LP	*p* Value
Age when FPC diagnosed, years			.631
Median	47.50 (interquartile range: 42.75–60.75)	54.50 (interquartile range: 46.75–57.75)	
Age when MM diagnosed, years			.190
Median	47.50 (interquartile range: 42.50–61.75)	56.50 (interquartile range: 53.00–60.75)	
Interval of the occurrence, months			.347
Median	15.90 (95%CI 12.30–19.50)	17.57 (95%CI 14.67–20.53)	
MM-OS, months			.968
Median	28.67 (95%CI 10.83–46.51)	43.73 (95%CI 33.40–54.06)	

**Figure 2.**
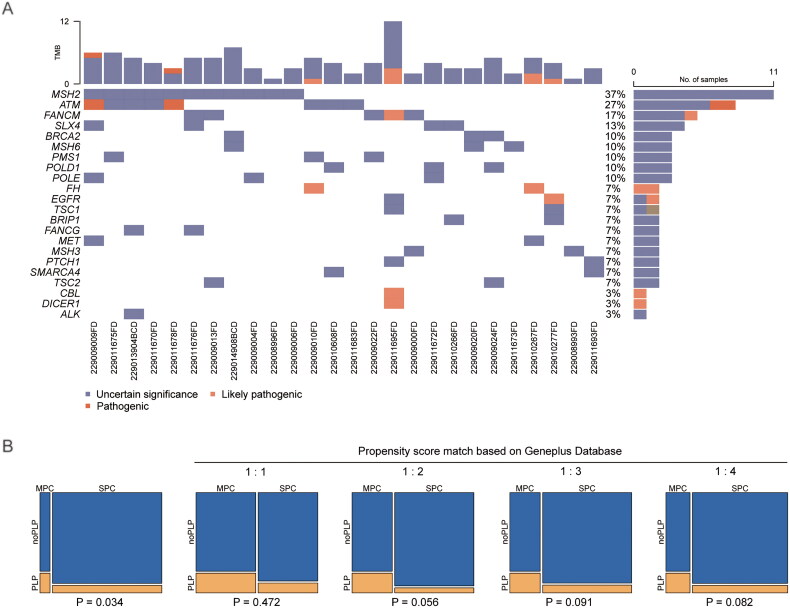
Difference of germline mutations in patients with melanoma and additional primary tumours. (A) Distributions of germline mutations in patients with melanoma and additional primary tumours. (B) Comparison of germline mutation rates between patients with MM and additional primary tumours and age-matched patients with single primary melanoma.

The P/LP germline mutated genes in the 30 patients were ATM (2/30), EGFR (1/30), FH (2/30), FANCM (1/30), CBL (1/30) and DICER1 (1/30), all pan-cancer germline pathogenic genes (Figure 2(A)). The MSH2 gene was found to be the most common altered gene, which observed in 11 of 30 patients, but the mutations did not qualify as P/LP. Gene mutations in the ATM gene were observed in 8/30 patients, seven patients had missense mutations and the remaining one had nonsense mutations. Mutations in FANCM were observed in 5/30 patients and were all missense mutations. Only two patients had ATM mutations that were P/LP mutations (one missense mutation and one nonsense mutation), and one patient had a FANCM gene mutation that was a P/LP mutation. Notably, there was a patient who had FANCM, CBL and DICER1 germline mutations at the same time. This patient had advanced unknown origin MM with multiple metastases. There was a BRAF mutation and a concomitant breast tumour that occurred with MMPC, without a family history of cancer and no other special clinical features. The patient died in a short time due to his advanced age and many underlying diseases. The details of gene P/LP mutation loci are shown in Table 5.

**Table 5.** Germline P/LP mutated genes and mutated loci in six patients.

Patients	Gene	Chromosome	Start position	End position	Reference	Tumour sequences	Variant type	Variant classification
1	ATM	11	108216485	108216486	TC	–	DEL	Frame shift deletion
2	FH	1	241676979	241676979	C	T	SNP	Missense mutation
3	FH	1	241680613	241680614	–	ATTTTGGATGAAGTAGGCTGTATTCATGAGTCACTTTAC	INS	Nonsense mutation
4	EGFR	7	55240695	55240695	G	A	SNP	Missense mutation
5	ATM	11	108142133	108142133	G	A	SNP	Nonsense mutation
6	CBL	11	119156167	119156168	–	GGGCAGTTTGTCTCTGGAGGGAC	INS	Frame shift insertion
6	FANCM	14	45658441	45658442	–	CAATGGTCCTGTTGTTTGTTCTTCTTAACAGTGCTT	INS	Nonsense mutation
6	DICER1	14	95566259	95566260	–	AAATGGGAAAAAGATGAAATGGTAAGTTTTTGTGTGTGTG	INS	Frame shift insertion

Another 327 MM single primary samples from the Geneplus database were included to explore the differences in germline background. Generally, compared to the MM single primary patients, patients with MM and additional primary tumours had a significantly higher germline mutation rate (25/327 vs. 6/30, 8.3% vs. 20.0%) (*p* = .034) (Figure 2(B)). The most often seen germline mutations in patients with single primary MM were in MUTYH (5/25), followed by mutations in MSH3 and ATM (both 3/25), but without hot spot variant types and special mutation loci.

### 3.3. Differences in somatic mutations in patients with MM and additional primary tumours

**Figure 3.**
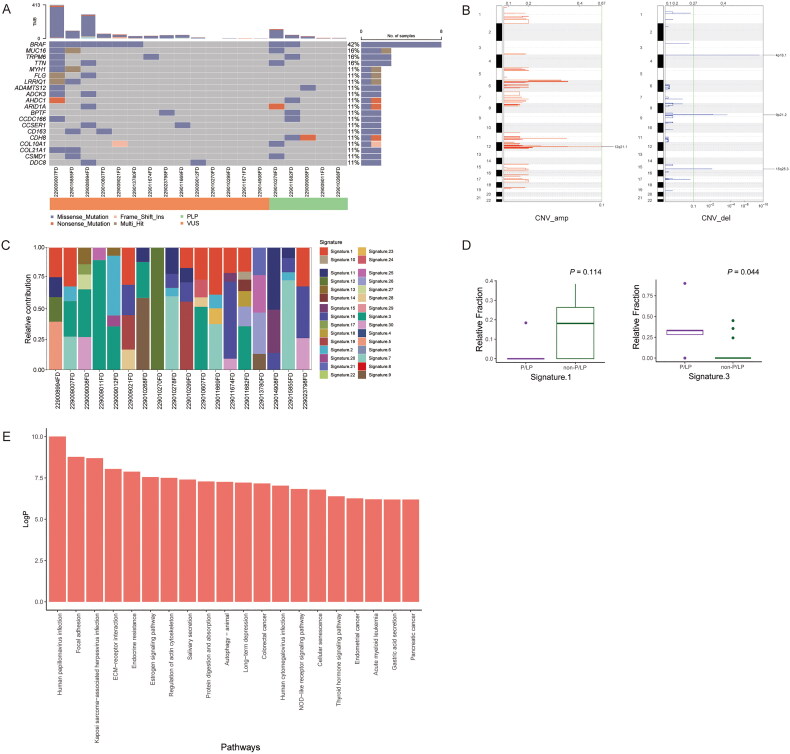
Difference of somatic mutations in 19 melanoma tissue samples. (A) Distributions of somatic mutations in 19 melanoma tissue samples. (B) The genomic CNV variation in 19 melanoma tissue samples. (C) Profiles of somatic COSMIC Signatures in 19 melanoma tissue samples. (D) Somatic COSMIC signature 3 was significantly higher in the melanoma tissue samples with P/LP germline mutations. (E) Enrichment genetic pathways in 19 melanoma tissue samples.

Whole exome sequencing was performed on the 19 samples of MM. The differences in CNV, COSMIC Signature, TMB, TNB, WGD, MSI, MSI score and homologous recombination repair (HRD) were determined. The BRAF gene had the highest mutation rate among the top 20 mutated genes in MM primary tumour samples (8/19, 42.11%) (Figure 3(A)). The mutation loci of the BRAF gene were altered as g.140453136 A > T in seven patients (87.5%), the corresponding amino acid loci altered as V600E. The remaining one patient had a rare, altered g.140453155 C > A in the BRAF gene. The corresponding amino acid loci were altered as D549N, but this patient had no P/LP germline mutations. The other high mutation rate genes were TTN (15.8%), IRPM6 (15.8%) and MUC16 (15.8%). It is noteworthy that MUC16 promotes tumour proliferation and has been found to be a novel anti-tumour target. There was one patient with a somatic MUC16 mutation accompanied by an EGFR germline mutation. Genomic CNVs in the MM primary tumour samples were mainly manifested as copy number amplifications on chromosome 12q21.1 and copy number deletions on chromosome 4p16.1, 9p21.2 and 15p25.3, as shown in Figure 3(B). Major amplification events on chromosome 12q21.1 included the genes TSPAN8 and LGR5. Prospective loss events on chromosome 4p16.1, 9p21.2 and 15p25.3 were located on gene TUSC1, which has been shown to be downregulated in lung tumorigenesis [27].

Overall, as shown in Table 6, in the 19 patients, the median scores of TMB were 1.05 (2.70 ± 5.61), and the median score of the HLALOH correction TMB was 0.66 (1.82 ± 3.03). There was one patient who had a HRD score of 42. The scores of HRD were not high in general, with the median of 9 (12.32 ± 14.63). But when grouped by shared properties, patients with MM as the first primary had a higher TMB and HLALOH correction TMB than those without MM as the first primary tumour (TMB median: 1.05 vs. 0.86, *p* = .982; HLALOH correction TMB median: 1.49 vs. 0.65, *p* = .492). The non-P/LP group had a higher TMB and HLALOH correction TMB. The non-P/LP vs. P/LP TMB median was 1.06 vs. 0.63, *p* = .754, and the HLALOH correction TMB median was 2.14 vs. 0.93, *p* = .622. Of interest was a trend towards a positive correlation between TMB and HRD in the non-P/LP group with the trend being reversed in the P/LP group. Furthermore, it was noticed that in patients with somatic BRAF gene mutations, the median HRD score was 13.5 (16.75 ± 14.86). While in patients without somatic BRAF gene mutations, more than half of the patients (6/10) displayed a HRD score of 0.0, and the average HRD score was 9.09 ± 14.26. WGD is another manifestation of genomic instability, and 3/19 patients had WGD. They all displayed MSI-H, but only one patient had a high TMB and somatic BRAF gene mutations while another one patient had germline mutations and somatic BRAF gene mutations.

**Table 6.** TMB, TNB, WGD, MSI, MSI score and HRD in the 19 patients with MM and additional primary tumours.

Patients	Group	TMB	HLALOH TMB	HLALOH	MSI_Score	MSI	WGD	HRD
1	MMFP	23.09	8.99	True	0.1895	MSI-H	WGD	0
2	Non-MMFP	11.74	10.97	True	0.0147	MSS	WGD	42
3	MMFP	0.63	0.63	False	0.0032	MSS	Non-WGD	33
4	Non-MMFP	0.03	0.03	False	0	MSS	Non-WGD	0
5	Non-MMFP	0.27	0.27	False	0	MSS	Non-WGD	2
6	MMFP	1.08	1.08	False	0.0054	MSS	Non-WGD	0
7	MMFP	0.03	0.03	False	0.0002	MSS	Non-WGD	0
8	MMFP	0	0	False	0.0001	MSS	Non-WGD	0
9	MMFP	3.53	2.91	True	0.0106	MSS	Non-WGD	14
10	MMFP	0.09	0.09	False	0.0011	MSS	Non-WGD	0
11	MMFP	1.92	1.92	False	0.0207	MSS	WGD	34
12	MMFP	1.35	1.35	False	0.0978	MSI-H	WGD	9
13	MMFP	0.21	0.21	True	0.0248	MSS	Non-WGD	0
14	MMFP	1.05	0.6	True	0.032	MSS	Non-WGD	18
15	Non-MMFP	1.44	1.03	True	0.1507	MSI-H	WGD	13
16	MMFP	0.66	0.66	False	0.0087	MSS	Non-WGD	19
17	Non-MMFP	0	0	False	0	MSS	Non-WGD	0
18	Non-MMFP	3	3	False	0.0072	MSS	WGD	12
19	MMFP	1.2	0.83	True	0.0029	MSS	WGD	38

The COSMIC signatures of 19 MM samples showed a prevalence of signature 1 and signature 3 (Figure 3(C)). Grouping these 19 MM samples by the properties of their P/LP germline mutations, signature 3 was significantly higher in the P/LP group than in the non-P/LP group (Figure 3(D)). Genetic pathway enrichment analysis was also performed on the mutated genes and significant enrichment was found in the cAMP signalling pathway, NOD-like receptor signalling pathway and ECM–receptor interaction pathway (Figure 3(E)).

## 4. Discussion

To our knowledge, this report is the first to show the clinical features, prognosis and genetic background of patients with MM and additional primary tumours in an Asian population. The incidence of secondary melanoma is quite high in European and American populations who first had cutaneous melanoma, with a rate of 51.40% [28]. So, in this study, secondary melanoma was excluded. The prevalence rate of MM and an additional primary tumour in this study was 4.74%, which is slightly higher than other MPMTs cohorts in Chinese populations [29–32]. This could be attributed to the advancements in medical care and increased awareness of and self-examination among the population in recent years. Notably, nearly 25% of patients were found with accompanying concomitant digestive tumours in this study, especially colorectal cancers, similar to the report of Yang et al. [33]. When MM was the first primary tumour, the median interval between tumour occurrence was less than 10 months, regardless of the family history of cancer. For patients with germline P/LP mutations, the interval time between second tumour occurrence was even shorter. Therefore, for patients with MM combined with additional primary tumours and expected survival of more than 10 months, the digestive examination for those patients, which is not limited to gastrointestinal endoscopy, ultrasound or abdominal CT, is recommended.

It was also observed that patients with MM as the first primary tumour had markedly the longer MM-OS. One possible reason might be the age of the melanoma onset in these patients, who were mostly below 60 years old, but more evidence was needed to better confirm. Interestingly, compared with patients without a family history of cancer, patients with a family history have significantly longer OS and MMS after MM diagnosis. This phenomenon has been reported in a few studies. Patients with a family history of hepatocellular carcinoma had a higher OS than those without [34]. Patients with a family history of SCC or lymphoma had double the risk of developing secondary tumours, but mortality was independent of family history [35,36]. Our study is also the first to analyse clinical features and prognosis in combination with germline mutations. The incidence of germline P/LP mutations is higher in patients with a family history of cancer, and the survival rate of patients with germline P/LP mutations is lower than that of patients without. Although there was no statistical significance, it reminds clinicians that complete follow-up of a family cancer history and detection of germline P/LP mutations will have potential predictive value for patient prognosis.

Hereditary and gene mutation predispositions are potential mechanisms of MPMTs. Germline CDKN2A mutations are frequently detected in multiple primary melanoma patients with family histories of melanoma and the most frequent somatic variants are the pathogenic BRAF V600E mutation [16]. In Asian populations, approximately 30% of patients with single primary melanoma have BRAF mutations, which is lower than the approximately 60% reported in European and American populations, and around 80% of BRAF mutations were known as V600E [37,38]. For the first time, this study reports that the incidence of BRAF mutations in patients with MM and additional primary tumours is significantly higher, at 42.11%, than in domestic single primary MM patients. The BRAF gene has the role of encoding serine/threonine kinases, being involved in the MAPK pathway [38]. BRAF mutations activate ERK and MAPK, leading to a series of cellular responses. Anti-BEAF targeted therapy, such as vemurafenib, dabrafenib and encorafenib, are available drugs. Particularly for ‘dabrafenib + trametinib’ combined therapy, it is reported that the median duration of progression-free survival can reach 11.1 months and 5-years overall survival (OS) rates were 34% [39]. In this study, a total of eight patients with BRAF mutations, four of them received anti-BRAF targeted therapy and their PFS were all more than 8 months, one patient withdrew from anti-BRAF targeted therapy due to financial reason, the other three patients chose surgery or immunotherapy. This means these patients can have more opportunities to benefit from target therapy.

Meanwhile, compared with the single primary melanoma cohort, the germline mutation rates are higher in patients with MM and additional primary tumours. The MSH2 gene had proved to be involved in DNA mismatch repair [40], but the mutations were not germline P/LP mutations in this study. A previous study had reported CDKN2A germline mutations as germline P/LP mutations in patients with multiple primary melanoma [41], while germline mutations of ATM and FANCM genes are for the first time reported as P/LP mutations in this study. ATM gene functions as a key initiator and coordinator of DNA double-strand break (DSB) repair and suppression of cancer at early stages [42]. A large investigation of predisposition variants in cancer demonstrated pathogenic or likely pathogenic germline mutations in 57 ATM genes were identified in 10,389 cancers distributed across at least 18 cancer types and notable pathogenic enrichment was observed in ovarian cancer (FDR <0.05), which suggested the carriers may have a higher risk of hereditary cancers [43]. Unfortunately, little was found on ATM germline mutations and multiple primary tumours. ATM mutant tumours show higher sensitivity to DNA damage drugs and immune checkpoint inhibitors. In this study, one patient with ATM germline mutations received PD-1 inhibitor for nearly 2 years, which initially offers possible treatment choice of these patients. The FANCM plays important roles in the stabilization and repair of replication fork, also involves in genome stability maintenance [44]. FANCM mutations were found in 12 different cancer types, including breast cancer, ovarian cancer and endometrial cancer, with rare frequency of variants for about 1.0% [11]. In this study, the patients with FANCM germline mutation had breast cancer as the first primary tumour and underwent endocrine therapy after surgery until development of the second primary tumour, melanoma. FANCM germline mutations with multiple primary tumours were also little studied, neither its treatment method. Therefore, more studies are needed on germline mutations and multiple primary tumours, as well as precision treatment strategy.

Seven patients in the study cohort displayed WGD, of which one was the cutaneous type, one was the mucosal type, two were the acral type and three were of unknown primary origin. In patients who had a single primary melanoma, WGD is most commonly associated with acral melanoma (71%), followed by mucosal (54%), cutaneous (54%) and unknown primary (19%) types [41]. In our study, there were three patients with WGD concomitant with MSI-H. One patient received PD-1 immunotherapy at the stage of rapid melanoma progression, but died two months later before the therapy was effective. One patient relapsed during postoperative adjuvant PD-1 treatment and switched to ‘dabrafenib + trametinib’ dual target therapy in combination with PD-1 after resistance and achieved a PR. The regime is still in use. The third patient did not receive PD-1 treatment or other therapies. Published reports of tumours with MSI suggest that these tumours tend not to have WGD events [45,46], which is contrary to our findings. It seems that more basic studies are needed exploring the potential mechanisms of any correlation between WGD and MSI-H.

Previous studies had performed extensive and in-depth sequencing analyses of gene rearrangements and CNVs in patients with single primary melanoma [41,47,48]. This study expands the scope of the melanoma population, completing CNV and HRD detections as well as COSMIC Signature analyses in patients with MM and additional primary tumours. The CNVs were mainly copy number amplifications in 12q21.1 and copy number deletions in 4p16.1, and chromosomal events showed up-regulation of TSPAN8 and LGR5 and down-regulation of TUSC1. Overexpressed TSPAN8 is correlated with an invasive phenotype and progression to metastasis in human cutaneous melanoma [49]. LGR5 is described as cancer stem cell marker in pan-cancer [50]. TUSC1 is a tumour suppressor gene that reduces tumour growth that is downregulated in lung cancer and has a role in tumorigenesis [51]. The COSMIC signatures reflect unique mutational features in cancer somatic mutations [52]. In this study, signature 1 correlates with age at cancer diagnosis, and signature 3 is associated with the failure of DNA DSB-repair by homologous recombination, which is predominant in patients with MM and additional primary tumours. The COSMIC signature 3 was significantly higher in P/LP patients than in the non-P/LP group. This corresponded with CNV changes with shorter survival time and shorter intervals between tumour occurrence in patients with MM and additional primary tumours, especially in patients who had germline P/LP mutations. Besides, cAMP signalling pathway, NOD-like receptor signalling pathway and ECM–receptor interaction pathway showed enrichment, which were found to be involved in cellular processes such as proliferation, differentiation, apoptosis, metabolism, immune response and inflammation [53–55].

It is still challenging for the treatment of multiple primary tumours. In Chinese guideline for diagnosis and treatment of cancer of multiple primaries, the general principle to make treatment regimens should follow: (1) decide the sequence of treatment according to the biological behaviour and clinical stage of each primary tumour; (2) first consider to treat tumours with high malignancy and advanced stage; (3) clarify the primary lesion of each metastasis [56]. Fortunately, in this study, most patients belonged to metachronous multiple primary tumours, one of the tumours was clinically cured with surgical and adjuvant treatment or underwent maintenance therapy. Therefore, it is only necessary to choose the best treatment options for the tumour in the moment. But for patients with synchronous multiple primary tumours, sequence of tumour development and clinical stages were the main considerations. In a total of 12 patients with SMPCs, six patients were diagnosed with advanced melanoma first; the second tumour was found in early stage, so the main treatment regimens focused on melanoma. The second primary tumour was surgically removed. For those patients who refused to receive surgery for the second primary tumours, treatment that worked for both tumours was used, such as immunotherapy combined with anti-vascular therapy. Four patients were diagnosed with melanoma first but in early stage, surgical and adjuvant treatment were chosen, as well as for the second primary tumours. One patient had thyroid cancer first and received surgical intervention; the following melanoma was treated according to melanoma guidelines. The last patient refused all kinds of treatment, only clinical follow-up was performed. Briefly, it is important to develop a treatment plan that first considers the highly life-threatening primary tumour, but also takes into account the treatment of other multiple primary tumours.

There are some limitations to this study. The overall number of patients with MM and additional primary tumours enrolled in this study was not satisfactory, and some patients’ tumour and normal tissues were not available. Thus, there may exist a bias in the subgroup analysis due to the small sample size.

In conclusion, this study is the first comprehensive analysis of the epidemiology, clinical features, prognosis and genetic differences of patients with MM and additional primary tumours, including prevalent age, subgroup analysis of the time sequence of tumour occurrence, cancer family histories, interval time of tumour occurrence, etc. Most patients with MMFP are younger than 60 years old. Digestive system tumours are the most common concomitant tumour type. Although tumour interval time is less than 10 months for patients first diagnosed with MM, the longer MSS time was observed for about 30 months. For the first time, germline and somatic gene sequencing were completed and they describe the genetic features in populations of patients with MM and additional primary tumours. Notably, patients with family histories of cancer had delayed onset of tumours and higher incidence of germline P/LP mutations. Germline P/LP mutations of ATM and FANCM genes are also the first time reported in patients with MM and additional primary tumours. Combined with the clinical characters, prognosis and genetic features, this study hopes to provide some clinical references and basis for the subsequent diagnosis and treatment of patients with MM and additional primary tumours.

## Supplementary Material

Supplementary table 5.docx

Supplementary method.docx

Supplementary table 1.docx

Supplementary table 4.docx

Supplementary table 3.docx

Supplementary table 2.docx

## Data Availability

The datasets used and/or analysed during the current study are available from the corresponding author upon reasonable request.
