# Supplementary material for: Differences of clinical features, prognosis and genetic mutations in Chinese patients with malignant melanoma and additional primary tumours
Source: Ann Med. 2025 May 3;57(1):2493769. doi: 10.1080/07853890.2025.2493769 (PMC12051608; doi:10.1080/07853890.2025.2493769)
Supplement: Supplementary table 5.docx [file IANN_A_2493769_SM7338.docx]

Supplementary table 5. Overview of clinical features in germline mutations patients with MM and additional primary tumors under conditions of tumor family histories (TFH).

| Variable | TFH group  (n, %) | non-TFH group  (n, %) | P -value |
| --- | --- | --- | --- |
| **Patients** | 5 | 25 |  |
| **P/LP germline mutations** |  |  |  |
| Yes | 2 | 4 | 1.000 |
| No | 3 | 21 |  |
| **First primary tumor** |  |  | 0.129 |
| Melanoma | 5 | 14 |  |
| Other | 0 | 11 |  |
| **Occurrence** |  |  | 0.254 |
| SMPC | 2 | 4 |  |
| MMPC | 3 | 21 |  |
| **Sites of Concomitant tumors (cases)** |  |  | 0.020 |
| Thyroid | 5 | 25 |  |
| Other | 1 | 20 |  |
