# Supplementary material for: Differences of clinical features, prognosis and genetic mutations in Chinese patients with malignant melanoma and additional primary tumours
Source: Ann Med. 2025 May 3;57(1):2493769. doi: 10.1080/07853890.2025.2493769 (PMC12051608; doi:10.1080/07853890.2025.2493769)
Supplement: Supplementary method.docx [file IANN_A_2493769_SM7337.docx]

**2.3 Genetic mutations**

**2.3.1 Whole exome sequencing**

DNA was extracted from fresh tissue samples that were obtained from the patient by surgical or needle biopsy before chemotherapy. QIAamp DNA Mini Kit (Qiagen) was used for DNA extraction and QIAamp DNA blood kit (Qiagen) was used for isolating germline genomic DNA from peripheral blood. The library was constructed following the instruction of SureSelect All Exon V5 Kit (Agilent, 5190-6209) and sequencing was performed on the HiSeq X platform. Fastp software was applied to remove low-quality and short reads and trim adapters from raw reads to get clean reads. Next, clean reads were aligned to the human reference genome (hg19) using the Sentieon-genomics pipeline (version section-genomics-201808). Read mapping was performed with bwa mem, duplicate reads were marked and removed with LocusCollector and Dedup algorithms. sQualCal was used for base quality score recalibration stage. After alignment, sorting, and deduplication, local realignment around indels on the BAM file was performed with realigner. Germline SNV and InDel were called with DNAscope after alignment and deduplication. ApplyVarCal was used for Variant Quality Score Recalibration (VQSR) stage.

**2.3.2 Somatic mutation calling**

SNVs and small InDels were called with GATK Mutect2 (version 4.1.4.1) software. A mutation was filtered out if it met any of the following criteria: (I) a mutation site was not a predefined hot spot or not in predefined hot regions that included EGFR exon 19/20, ERBB2 exon 20, and MET exon 14, and the variant allele frequency (VAF) of the mutation was less than 0.03. (II) a mutation occurred frequently in most human cancers, but it was not a true mutation that caused cancer or occurred often in normal tissues, and VAF was less than 0.05. (III) a mutation appeared in more than 1% of the population in the 1000G (1000 Genomes Project, version phase 3), 1000G-EAS (1000G East Asian Project), ExAC database (The Exome Aggregation Consortium, version 0.2), or ExAC-EAS (ExAC East Asian). (IV) a mutation was in the predefined blacklist database. Then TMB was calculated as the number of non-synonymous mutations per megabase of coding region. The coding region was defined as the union of all exons from RefSeq genes. MSI was calculated using a published MSI sensor tool (v0.2).

**2.3.3 tumor neoantigen burden (TNB)**

TNB was defined as the number of predicted neoantigens per megabase of tumor genome. And TNB was calculated using pVACseq, a computational tool for predicting and prioritizing potential neoantigens from tumor genomic data. pVACseq supports the identification of neoantigens from different mutational mechanisms, including SNVs, indels and gene fusions. pVACseq also integrates multiple data sources, such as mutant allele expression level, peptide-MHC binding affinity, clonality and subclonality of mutations, to rank and filter the predicted peptides.

**2.3.3 Copy number variation**

Somatic CNV was identified with FACETS (v0.5.11). CNV gains were defined as segments with copy number/ploidy > log2(2.8/2), while CNV losses were segments with copy number/ploidy < log2(1.4/2). The CCF of each copy number event was calculated as dividing the cf.em column by the purity of the sample. Sometimes the cf.em value might be slightly higher than the purity value due to noise, in which case the CCF estimates would be rounded to 1.0 (100%). CCF greater than 1.0 would be set to 1.0. CCF greater than 0.7 was considered as clonal CNV. Otherwise, it was a subclonal CNV. GISTIC (Genomic Identification of Significant Targets in Cancer) analysis was performed to identify significant arm-level and focal-level CNV.

**2.3.4 Mutational signature analysis**

Mutational signatures are characteristic combinations of mutation types arising from specific mutagenesis processes. These processes include DNA replication infidelity, exogenous and endogenous genotoxin exposures, defective DNA repair pathways, and DNA enzymatic editing. A mutational signature is the outcome of a mutagenic process comprising some form of DNA damage, subsequently acted upon by DNA repair and/or replicative machinery. Mutational signature analysis can provide insights into the biological mechanisms involved in carcinogenesis and normal somatic mutagenesis. Mutational signature analysis was conducted using an R package *deconstructSigs* (version 1.8.0). Signatures were defined according to the Catalogue of Somatic Mutations in Cancer.

(COSMIC; version 2; <https://cancer.sanger.ac.uk/signatures/signatures_v2>).

**2.3.5 Tumor purity, ploidy, WGD, and HRD**

ABSOLUTE (v1.2) was used to estimate the tumor purity, ploidy, and whole-genome doubling (WGD) for each sample based on the copy number profiles. HRD was calculated using scarHRD package, which determines the levels of genomic scars (telomeric allelic imbalance, loss of heterozygosity, and number of large-scale transitions) based on NGS data. The HRD score was defined as the sum of these three genomic scar scores.

**2.3.6 Germline variant analysis and annotation**

The Illumina analysis pipeline (CASAVA 1.8) was used for base-calling to convert raw sequencing data into nucleotide sequences. Low-quality data was removed and each barcoded dataset was separated. Reads were mapped to the reference genome GRCh37/hg19 using Burrows–Wheeler Aligner. GATK (Version 3.6) (haplotype caller in single-sample mode with duplicate and unmapped reads removed using defaulted parameters) was used to detect SNVs and small indels from germline DNA samples extracted from blood. Variants in 94 genes (selected from Genetic Testing Registry46 (GTR, www.ncbi.nlm.nih.gov/gtr/) and NCCN Genetic/Familial High-Risk Assessment guidelines) were included for further annotation. Variants were annotated using InterVar software, which implements the ACMG/AMP 2015 guidelines for clinical interpretation of sequence variants. InterVar assigns a clinical significance to variants as pathogenic, likely pathogenic, uncertain signiﬁcance, likely benign or benign based on various types of evidence classiﬁed as PVS1 (pathogenic very strong 1), PS2 (pathogenic strong 2), PS3 (pathogenic strong 3), PM6 (pathogenic moderate 6) and BS3 (benign strong 3) in ACMG guideline.

**2.3.7 HLA genotyping and HLALOH identification**

OptiType, an HLA genotyping algorithm based on integer linear programming, was used to predict human leukocyte antigen (HLA) genotypes from NGS data by simultaneously selecting all minor and major HLA-I alleles from germline DNA samples extracted from blood. HLALOH repository, a computational tool to evaluate HLA loss using next-generation sequencing data and HLA genotyping, was used to identify HLALOH (loss of heterozygosity in human leukocyte antigen). We also calculated the HLA-corrected TMB as a measure of tumor immunogenicity and predictor of immune checkpoint inhibitor efficacy.
