# Supplementary material for: Differences of clinical features, prognosis and genetic mutations in Chinese patients with malignant melanoma and additional primary tumours
Source: Ann Med. 2025 May 3;57(1):2493769. doi: 10.1080/07853890.2025.2493769 (PMC12051608; doi:10.1080/07853890.2025.2493769)
Supplement: Supplementary table 1.docx [file IANN_A_2493769_SM7336.docx]

Supplementary table 1. Overview of clinical features in patients with MM and additional primary tumors under the conditions of SMPC & MMPC.

| Variable | Overall  (n) | SMPC group  (n, %) | MMPC group  (n, %) | P -value |
| --- | --- | --- | --- | --- |
| **Patients** | 58 | 12 | 46 |  |
| **Gender** |  |  |  | 0.348 |
| Male | 27 | 5 | 12 |  |
| Female | 31 | 7 | 34 |  |
| **Age when FPC diagnosed, y** |  |  |  | 0.732 |
| ＜60 | 35 | 6 | 29 |  |
| 60-80 | 22 | 6 | 16 |  |
| ＞80 | 1 | 0 | 1 |  |
| **Age when MM diagnosed, y** |  |  |  | 0.661 |
| ＜60 | 28 | 6 | 22 |  |
| 60-80 | 27 | 6 | 21 |  |
| ＞80 | 3 | 0 | 3 |  |
| **Number of cancer type** |  |  |  | 0.076 |
| 2 | 52 | 12 | 40 |  |
| 3 | 5 | 0 | 5 |  |
| 4 | 1 | 0 | 1 |  |
| **Order of tumor occurrence** |  |  |  | 0.009 |
| Melanoma first | 28 | 10 | 18 |  |
| Other tumors first | 30 | 2 | 28 |  |
| **Primary location of MM** |  |  |  | 0.839 |
| Cutaneous | 10 | 1 | 9 |  |
| Acral | 26 | 6 | 20 |  |
| Mucosal | 13 | 3 | 10 |  |
| Unknown primary | 9 | 2 | 7 |  |
| **MM clinical stage** |  |  |  | 0.235 |
| I | 25 | 2 | 23 |  |
| II | 7 | 3 | 4 |  |
| III | 9 | 3 | 6 |  |
| IV | 17 | 4 | 13 |  |
| **BRAF mutation** |  |  |  | / |
| V600E mutation | 8 | 0 | 8 |  |
| Wild type | 17 | 7 | 10 |  |
| Undetected | 33 | 5 | 28 |  |
| **Treatment of melanoma** |  |  |  | 1.000 |
| Surgery | 46 | 9 | 37 |  |
| Chemotherapy | 9 | 1 | 8 |  |
| Target /Immunological | 27 | 9 | 18 |  |
| **Sites of Concomitant tumors** |  |  |  | 0.679 |
| head and neck | 5 | 1 | 4 |  |
| Thyroid | 11 | 3 | 8 |  |
| Respiratory system | 13 | 2 | 11 |  |
| Digestive system | 21 | 3 | 18 |  |
| Urogenital System | 10 | 0 | 10 |  |
| Breast | 2 | 0 | 2 |  |
| Others | 2 | 2 | 0 |  |
| **Status** |  |  |  | / |
| Alive | 25 | 6 | 19 |  |
| Dead | 33 | 6 | 27 |  |
| **Tumor family history** |  |  |  | 1.00 |
| No | 46 | 9 | 37 |  |
| Yes | 12 | 3 | 9 |  |
| Genetic risk classification |  |  |  |  |
| Extremely high | 4 | 1 | 3 |  |
| High | 3 | 2 | 1 |  |
| Median | 5 | 0 | 5 |  |
