# Supplementary material for: Differences of clinical features, prognosis and genetic mutations in Chinese patients with malignant melanoma and additional primary tumours
Source: Ann Med. 2025 May 3;57(1):2493769. doi: 10.1080/07853890.2025.2493769 (PMC12051608; doi:10.1080/07853890.2025.2493769)
Supplement: Supplementary table 3.docx [file IANN_A_2493769_SM7334.docx]

Supplementary table 3. Overview of clinical features in patients with or without tumor family histories (TFH).

| Variable | Overall  (n) | With TFH  (n) | Without TFH  (n) | P -value |
| --- | --- | --- | --- | --- |
| **Patients** | 58 | 12 | 46 |  |
| **Gender** |  |  |  | 0.348 |
| Male | 27 | 4 | 13 |  |
| Female | 31 | 8 | 23 |  |
| **Status** |  |  |  | 0.101 |
| Alive | 25 | 9 | 16 |  |
| Dead | 33 | 3 | 30 |  |
| **Age when FPC diagnosed, y** |  |  |  | 0.304 |
| ＜60 | 35 | 6 | 29 |  |
| 60-80 | 22 | 6 | 16 |  |
| ＞80 | 1 | 0 | 1 |  |
| **Age when MM diagnosed, y** |  |  |  | 0.899 |
| ＜60 | 28 | 4 | 24 |  |
| 60-80 | 27 | 6 | 21 |  |
| ＞80 | 3 | 1 | 2 |  |
| **Number of cancer type** |  |  |  | 0.481 |
| 2 | 52 | 10 | 42 |  |
| 3 | 5 | 2 | 3 |  |
| 4 | 1 | 0 | 1 |  |
| **Occurrence** |  |  |  | 0.401 |
| SMPC | 12 | 3 | 9 |  |
| MMPC | 46 | 9 | 37 |  |
| **Primary location of MM** |  |  |  | 0.839 |
| Cutaneous | 10 | 1 | 9 |  |
| Acral | 26 | 8 | 18 |  |
| Mucosal | 13 | 2 | 11 |  |
| Unknown primary | 9 | 1 | 8 |  |
| **MM clinical stage** |  |  |  | 0.215 |
| I | 25 | 4 | 21 |  |
| II | 7 | 1 | 5 |  |
| III | 9 | 5 | 4 |  |
| IV | 17 | 2 | 15 |  |
| **BRAF mutation** |  |  |  | 0.860 |
| V600E mutation | 8 | 2 | 6 |  |
| Wild type | 17 | 5 | 12 |  |
| Undetected | 33 | 5 | 28 |  |
| **Treatment of melanoma** |  |  |  | 1 |
| Surgery | 46 | 12 | 34 |  |
| Chemotherapy | 9 | 1 | 8 |  |
| Target /Immunological | 27 | 9 | 18 |  |
| **Sites of Concomitant tumors** |  |  |  | 0.425 |
| head and neck | 5 | 0 | 5 |  |
| Thyroid | 11 | 4 | 7 |  |
| Respiratory system | 13 | 4 | 9 |  |
| Digestive system | 21 | 4 | 17 |  |
| Urogenital System | 10 | 2 | 8 |  |
| Breast | 2 | 0 | 2 |  |
| Others | 2 | 1 | 1 |  |
| **Death reason** |  |  |  | 1 |
| Melanoma | 20 | 2 | 18 |  |
| Other | 13 | 1 | 12 |  |
| **Family history** |  |  |  | / |
| No | 46 | 0 | 46 |  |
| Yes | 12 | 12 | 0 |  |
| **First-generation family** | 8 | 8 | 0 |  |
| **Second- generation family** | 2 | 2 | 0 |  |
| **Both** | 2 | 2 | 0 |  |
| **Genetic risk classification** |  |  |  |  |
| Extremely high | 4 | 4 | 0 |  |
| High | 2 | 2 | 0 |  |
| Median | 4 | 4 | 0 |  |
| Low | 2 | 2 | 0 |  |
|  |  |  |  |  |
