# Supplementary material for: Differences of clinical features, prognosis and genetic mutations in Chinese patients with malignant melanoma and additional primary tumours
Source: Ann Med. 2025 May 3;57(1):2493769. doi: 10.1080/07853890.2025.2493769 (PMC12051608; doi:10.1080/07853890.2025.2493769)
Supplement: Supplementary table 2.docx [file IANN_A_2493769_SM7333.docx]

Supplementary table 2. Differences in age, interval time and survival in patients with MM and additional primary tumors under the conditions of SMPC & MMPC.

|  | Overall | SMPC | MMPC | P-value |
| --- | --- | --- | --- | --- |
| **Age when FPC diagnosed, y** |  |  |  | 0.323 |
| Median | 57.00  (interquartile range: 50.75 to 65.25) | 61.00 | 56.50 |  |
| **Age when MM diagnosed, y** |  |  |  | 0.863 |
| Median | 60.00  (interquartile range: 53.00 to 67.25) | 61.00 | 60.00 |  |
| **Interval of the occurrence, m** |  |  |  | 0.001 |
| Median | 29.84  (interquartile range: 6.53 to 78.07) | 0.57 | 45.37 |  |
| **MM-OS, m** |  |  |  | 0.552 |
| Median | 43.03 | 39.50 | 46.33 |  |
| **MSS, m** |  |  |  | 0.847 |
| Median | 12.27 | 11.57 | 12.27 |  |
|  |  |  |  |  |
